# Distance to care, care seeking and child mortality in rural Burkina Faso: findings from a population‐based cross‐sectional survey

**DOI:** 10.1111/tmi.13170

**Published:** 2018-11-18

**Authors:** S. Sarrassat, N. Meda, H. Badolo, M. Ouedraogo, H. Somé, S. Cousens

**Affiliations:** ^1^ Centre for Maternal Adolescent Reproductive and Child Health (MARCH) London School of Hygiene and Tropical Medicine London UK; ^2^ Centre Muraz Bobo Dioulasso Burkina Faso; ^3^ Africsanté Bobo Dioulasso Burkina Faso

**Keywords:** distance to care, care seeking, under‐five child mortality, neonatal mortality, Burkina Faso, distance aux soins, recherche de soins, mortalité des moins de 5 ans, mortalité néonatale, Burkina Faso

## Abstract

**Objective:**

Although distance has been identified as an important barrier to care, evidence for an effect of distance to care on child mortality is inconsistent. We investigated the association of distance to care with self‐reported care seeking behaviours, neonatal and post‐neonatal under‐five child mortality in rural areas of Burkina Faso.

**Methods:**

We performed a cross‐sectional survey in 14 rural areas from November 2014 to March 2015. About 100 000 women were interviewed on their pregnancy history and about 5000 mothers were interviewed on their care seeking behaviours. Euclidean distances to the closest facility were calculated. Mixed‐effects logistic and Poisson regressions were used respectively to compute odds ratios for care seeking behaviours and rate ratios for child mortality during the 5 years prior to the survey.

**Results:**

Thirty per cent of the children lived more than 7 km from a facility. After controlling for confounding factors, there was a strong evidence of a decreasing trend in care seeking with increasing distance to care (*P* ≤ 0.005). There was evidence for an increasing trend in early neonatal mortality with increasing distance to care (*P* = 0.028), but not for late neonatal mortality (*P* = 0.479) and post‐neonatal under‐five child mortality (*P* = 0.488). In their first week of life, neonates living 7 km or more from a facility had an 18% higher mortality rate than neonates living within 2 km of a facility (RR = 1.18; 95%CI 1.00, 1.39; *P* = 0.056). In the late neonatal period, despite the lack of evidence for an association of mortality with distance, it is noteworthy that rate ratios were consistent with a trend and similar to or larger than estimates in early neonatal mortality. In this period, neonates living 7 km or more from a facility had an 18% higher mortality rate than neonates living within 2 km of a facility (RR = 1.18; 95%CI 0.92, 1.52; *P* = 0.202). Thus, the lack of evidence may reflect lower power due to fewer deaths rather than a weaker association.

**Conclusion:**

While better geographic access to care is strongly associated with increased care seeking in rural Burkina Faso, the impact on child mortality appears to be marginal. This suggests that, in addition to improving access to services, attention needs to be paid to quality of those services.

## Abstract (fr)

**Objectif:**

Bien que la distance ait été identifiée comme un obstacle important aux soins, les preuves d'un effet de la distance aux soins sur la mortalité infantile sont incohérentes. Nous avons étudié l'association entre la distance aux soins et les comportements de recherche de soins auto déclarés, ainsi que la mortalité néonatale et post‐néonatale chez les moins de cinq ans dans les zones rurales du Burkina Faso.

**Méthodes:**

Enquête transversale dans 14 zones rurales, de novembre 2014 à mars 2015. Environ 100.000 femmes ont été interrogées sur leurs antécédents de grossesse et environ 5.000 mères sur leurs comportements en matière de recherche de soins. Les distances euclidiennes à l’établissement le plus proche ont été calculées. Les régressions logistiques à effets mixtes et les régressions de Poisson ont été utilisées respectivement pour calculer les rapports de cotes (odds ratios) des comportements de recherche de soins et les ratios des taux de mortalité infantile au cours des cinq années précédant l'enquête.

**Résultats:**

30% des enfants vivaient à plus de 7 km d'un établissement. Après ajustement des facteurs confusionnels, il y avait de fortes preuves d'une tendance à la baisse de la recherche de soins avec une distance aux soins croissante (*p* ≤ 0,005). Il y avait des preuves d'une tendance à la hausse de la mortalité néonatale précoce avec l'augmentation de la distance aux soins (*p* = 0,028), mais pas pour la mortalité néonatale tardive (p = 0,479) ni pour la mortalité post‐néonatale des enfants de moins de cinq ans (p = 0,488). Au cours de leur première semaine de vie, les nouveau‐nés vivant à 7 km ou plus d'un établissement avaient un taux de mortalité de 18% supérieur à celui des nouveau‐nés vivant à moins de 2 km d'un établissement (RR = 1,18; IC95%: 1,00 ‐ 1,39; *p* = 0,056). A la fin de la période néonatale, malgré l'absence de preuves d'un lien entre la mortalité et la distance, il convient de noter que les rapports de taux étaient cohérents avec une tendance et similaires ou supérieurs aux estimations de la mortalité néonatale précoce. Au cours de cette période, les nouveau‐nés vivant à 7 km ou plus d'un établissement avaient un taux de mortalité supérieur de 18% à celui des nouveau‐nés vivant à moins de 2 km d'un établissement (RR=1,18; IC95%: 0,92 ‐ 1,52; *p* = 0,202). Ainsi, le manque de preuves pourrait refléter une puissance plus faible due à moins de décès plutôt qu’à une association plus faible.

**Conclusion:**

Alors qu'un meilleur accès géographique aux soins est étroitement associé à une augmentation de la recherche de soins dans les zones rurales du Burkina Faso, l'impact sur la mortalité infantile semble être marginal. Cela suggère qu'en plus d'améliorer l'accès aux services, il convient de prêter attention à la qualité de ces services.

## Introduction

Despite a large reduction in under‐five deaths worldwide from 1990 to 2015, scenario‐based projections suggest that about two‐thirds of all sub‐Saharan African countries will need to accelerate their progress to achieve the Sustainable Development Goal (SDG) target of 25 or fewer under‐five deaths per 1000 live births by 2030 1. An analysis of five countries using the Lives Saved Tool (LiST) estimated that increases in coverage of obstetric and newborn care accounted for 33% to 44% of averted neonatal deaths depending on country, while increased coverage of measures to prevent and treat infections accounted respectively for 28% to 72% and for 2% to 10% of averted post‐neonatal under‐five deaths 2. Poor coverage of effective interventions for preventing child deaths has been attributed to weaknesses in both provision of and demand for services 3, 4. Increased provision of services through better access to good‐quality care could therefore potentially reduce child mortality.

Access to care depends on a wide range of factors including environmental factors and population characteristics 5. Although distance has been identified as an important barrier to health care access (‘distance decay effect’) 6, 7, 8, 9, 10, 11, evidence for an effect of distance to care on child mortality is inconclusive and at times contradictory. While a review, based on eight studies in sub‐Saharan Africa, concluded that there was no robust evidence of an association between distance to care and child mortality 5, subsequent meta‐analyses have reported evidence of increasing mortality risk with decreasing access to care in neonates, infants` and under‐five children 11, 12. Here we report on the association of distance to facility with care seeking behaviours, neonatal and post‐neonatal under‐five child mortality across 14 rural areas of Burkina Faso, with low health service density.

## Methods

### Setting

Burkina Faso is a landlocked country in West Africa with a population in 2015 estimated at 18 106 000 inhabitants (https://esa.un.org/unpd/wpp). About three‐quarters of the population live in rural areas, largely depend on subsistence agriculture, and about half of the population live below the poverty line 13. Since 1990, the under‐five mortality rate has declined from an estimated 202 deaths per 1000 live births to 89 in 2015 1.

The government is the main health service provider, managing 83% of the facilities within the country in 2014 14. The country is divided into 13 regions and 63 health districts each with one district or regional hospital. In 2014, the public health system included four Centres Hospitaliers Universitaires (CHU), nine Centres Hospitaliers Regionaux (CHR), 47 Centres Medicaux avec Antenne Chriurgicale (CMA) and 1859 primary health facilities, corresponding to about one hospital per 300 000 inhabitants and one primary facility per 10 000 inhabitants. In rural areas, primary health facilities, run by nurses, are the most common point of care and provide a basic package of outpatient services. At the time of the study, free antenatal care (ANC) and subsidised childbirth and emergency obstetric and neonatal care (EmONC) were provided in all public facilities (basic EmONC in first level facilities, comprehensive EmONC in second and third level facilities). Details of services provided and their availability, as reported by the 2014 Service Availability and Readiness Assessment (SARA), are given in Table 1 14. At the community level, case management of malaria with artemisinin‐based combination therapy (ACT) was scaled up in 2010 15, and late 2013, the Micronutrient Initiative, together with the Ministry of Health (MoH), launched the Zinc Alliance for Child Health (ZACH), with the aim of scaling up oral rehydration salt (ORS) and zinc for treating childhood diarrhoea.

**Table 1:** Services, trainings, and essential medicines available in health facilities (%)

	Health facilities^a^		
1st level	2nd level	3rd level
Antenatal care (ANC)			
Iron and folic acid supplementation	90	72	89
Intermittent preventive treatment (IPT)	91	71	89
Tetanus toxoid vaccination	89	55	44
Blood pressure monitoring	90	72	89
Basic emergency obstetric and neonatal care (BEmONC)			
Parenteral antibiotics	78	71	89
Parenteral oxytocin	80	71	89
Parenteral magnesium sulphate	17	64	89
Assisted vaginal delivery	89	70	89
Manual removal of placenta	71	70	83
Removal of retained products	12	67	83
Neonatal resuscitation (with bag and mask)	27	65	94
Comprehensive emergency obstetric and neonatal care (CEmONC)			
Caesarean section	0	60	89
Blood transfusion	0	62	94
Training (two past years)			
Health workers trained in ANC	56	73	56
Health workers trained in essential obstetric care	61	86	76
Health workers trained in neonatal resuscitation	41	83	100
Health workers trained in IMCI	63	58	39
Essential medicines (availability observed or reported)			
ACT tablet	91	65	78
Amoxicillin dispersible tablets or syrup	83	60	67
Cotrimoxazole syrup	85	65	67
ORS	82	54	72
Parenteral ampicillin	90	70	83
Parenteral or rectal artesunate	33	45	61
Parenteral gentamicin	81	72	83
Parenteral oxytocin	96	97	88
Parenteral magnesium sulphate	23	80	88
Sulphadoxine/Pyrimethamine tablet	66	39	75

Source: 2014 Service Availability and Readiness Assessment (SARA).

a 1st level: Primary health facilities; 2nd level: Centres Medicaux avec Antenne Chriurgicale (CMA); 3rd level: Centres Hospitaliers Universitaires (CHU), Centres Hospitaliers Regionaux (CHR).

### Study design

We performed a cross‐sectional household survey in 14 clusters across the country from November 2014 to March 2015. Clusters were selected for inclusion in a randomised trial evaluating the effect of a radio campaign on family behaviours and child mortality 16, 17. Each cluster was centred around a town with a community FM radio station and included approximately 40 000 inhabitants with limited access to television. The latter was achieved by excluding the communities living in and within 5 km of towns, villages with electricity or with more than 5000 inhabitants. With the exception of Kantchari cluster, the study population had access to a regional or district hospital in the town located at the centre of the cluster.

In all villages, a census of households was performed with Geographical Positioning System (GPS) co‐ordinates recorded. All women of the reproductive age were interviewed on their pregnancy history and about 5000 mothers with at least one under‐five child was selected, using systematic random sampling, to be interviewed on their care seeking behaviours (contraception uptake, ANC attendance and place of delivery for the last pregnancy of more than 6 months duration, care seeking for child's fever, cough, fast/difficult breathing, diarrhoea in the 2 weeks prior to interview). Sample size calculations for evaluation purposes have been reported elsewhere 16, 17.

A list of 1564 public health facilities located in or near the 14 clusters included in the study was obtained from the Burkina Faso MoH along with their GPS co‐ordinates.

Prior to the survey, fieldworkers received 2 weeks training. The data collection involved 84 fieldworkers who were deployed across the 14 clusters. Questionnaires were programmed into Personal Digital Assistants (PDA) and interviews were performed in local languages. Re‐interviews were requested for 7% of women due to incompleteness and/or inconsistencies, and all re‐interviews were completed.

### Ethics

The study was approved by the ethics committees of the Burkina Faso MoH and the London School of Hygiene and Tropical Medicine. Women recorded their consent to participate in the survey on the PDA. This study was embedded in a randomised trial evaluating the effect of a radio campaign on family behaviours and child mortality 16, 17. The trial was registered at ClinicalTrial.gov (Identifier: NCT01517230).

### Analyses

Mortality analyses were performed using the survival‐time family of commands in Stata 13.1. The primary outcomes of interest were neonatal (0 to 27 days of life) and post‐neonatal under‐five child (1 to 59 months of life) mortality. Neonatal mortality was further broken down into early (0 to 6 days of life) and late neonatal mortality (7 to 27 days of life). The period under study was restricted to the 5 years prior to the first month of the survey; i.e. from November 2009 to October 2014. The proportion of missing months of birth was low, at 2.6%, and these were randomly imputed according to the DHS method 18.

Rate ratios for child mortality were computed using a mixed‐effects Poisson regression, with cluster fitted as a fixed effect and village fitted as random effect. Controlling for cluster accounted for any effect of the radio campaign on child mortality, though the evaluation did not detect an effect 17.

Euclidean distances from each household to the closest public health facility (all types) and to the closest public hospital (CHU, CHR or CMA) were calculated in kilometres. Missing GPS co‐ordinates (5%) were replaced by the village mean distance. Distance to the closest facility was grouped into four categories (<2 km, 2–4 km, 4–7 km and >7 km), corresponding approximately to quartiles of the population. In Kantchari cluster, nearly all the children (99.6%) lived 30 km or more away from a hospital. Analyses of distance to the closest hospital therefore excluded Kantchari cluster, and distance to the closest hospital was grouped into three categories (<10 km, 10–20 km and >20 km), corresponding approximately to tertiles.

The model included the household wealth quintile, mother's age at the child's birth, child's gender and age (split into the following bands: <1, 1–5, 6–11, 12–17, 18–23, 24–35 and 36–59 months old) as forced variables. Other covariates associated with both the child mortality and distance to the closest facility or hospital were included as potential confounding factors: at the mother's level, ethnicity, religion, education level, marital status and duration of residence in the village; at the child level, birth order, preceding and succeeding birth interval lengths. The household wealth quintile was generated from a household wealth index computed from the first component of a polychoric principal component analysis of 22 household assets and goods 19.

Care seeking behaviours included use of a modern contraceptive method, attendance at four or more ANC visits, facility delivery and care seeking for childhood illness. Modern contraception was defined as oral contraception, intra‐uterine device (IUD), implant, injectable, sterilisation, diaphragm or spermicidal agents.

The analysis of the association between distance to the closest facility and care seeking behaviours used mixed‐effects logistic regression with cluster as a fixed effect and village as a random effect. The evaluation of the radio campaign found some evidence for an effect on care seeking behaviours 16, 17 and controlling for cluster will have accounted for this. The model included the household wealth quintile, mother's age at interview, child's gender and age at interview as forced variables. Mother's ethnicity, religion, education level, marital status, duration of residence in the village, and parity (number of stillbirths and live births) were included as potential confounders.

Effect modification by household wealth tertile and mother's school attendance was assessed by fitting a linear interaction term between the factor of interest and the distance to facility in the final model in order to investigate whether the association of distance to care with either self‐reported care seeking behaviours or child mortality differed by socio‐economic status and maternal education.

## Results

The survey identified a total of 108 151 women as resident in study villages, of whom 104 303 were present at the time of the survey (3.6% absent), 104 219 gave their consent to be interviewed (<1% refusals), 102 684 were aged 15 to 49 years old and provided information on 359 081 live births. All analyses were restricted to the 194 293 children under‐five who contributed person‐time at risk to the study period. A total of 12 841 under‐five deaths were recorded, of which 20% (2612) occurred in the first 28 days of life. The neonatal (0 to 27 days), post‐neonatal (1 to 59 months) and under‐five (0 to 59 months) child mortality risks were estimated at 23.4 per 1000 live births, 97.8 per 1000 children and 118.9 per 1000 live births respectively. In the neonatal period, 66% (1732) of neonatal deaths occurred in the first week of life (0 to 6 days) and the early (0 to 6 days) and late (7 to 27 days) neonatal mortality risks were estimated at 15.5 and 8.0 per 1000 live births respectively.

In total, 5657 mothers of children under 5 years were selected to be interviewed on their behaviours. All consented. Of these, 5110 women were not pregnant at the time of interview, of whom 27% reported current use of modern contraception. Forty‐nine per cent of all women reported four or more ANC visits and 80% reported having given birth in a facility for their last pregnancy. Fifty‐one per cent of mothers with a sick child in the past 2 weeks reported having sought care in a facility. Care seeking in a hospital was rare (reported by 6% or fewer women).

Cluster‐adjusted associations of socio‐demographic characteristics with under‐five child mortality, neonatal mortality, post‐neonatal under‐five child mortality and self‐reported care seeking behaviours are shown in Table 2 and Tables S1–S6.

**Table 2:** Cluster‐adjusted associations with under‐five child mortality

		Number of deaths	Person‐years	Rate per 1000 person‐years	95% CI		Cluster‐adjusted analysis				
							Rate ratio	95% CI		*P* value	Likelihood ratio test *P* value
Household wealth quintile	Poorest	2270	70 546	**32.2**	26.4	39.6	**1**	‐	‐	‐	<0.001
	2nd quintile	2427	80 784	**30.0**	25.0	36.4	**0.97**	0.91	1.02	0.240	
	3rd quintile	2469	91 575	**27.0**	22.3	33.0	**0.88**	0.83	0.93	<0.001	
	4th quintile	2564	101 669	**25.2**	21.5	29.7	**0.85**	0.80	0.90	<0.001	
	Least poor	2978	120 472	**24.7**	20.6	29.8	**0.83**	0.78	0.88	<0.001	
Mother's age at birth (years)	14–20	3216	103 546	**31.1**	26.2	37.3	**1.24**	1.17	1.30	<0.001	<0.001
	21–25	3238	131114	**24.7**	20.8	29.7	**1.00**	0.95	1.05	0.998	
	26–30	2629	107 212	**24.5**	20.5	29.7	**1**	‐	‐	‐	
	31–35	2055	75 032	**27.4**	23.3	32.4	**1.13**	1.07	1.20	<0.001	
	34–49	1697	53 599	**31.7**	26.7	37.8	**1.32**	1.24	1.41	<0.001	
Mother's ethnicity^a^										<0.001	
Mother's religion	Muslim	7209	264024	**27.3**	22.1	34.0	**1.08**	1.03	1.14	0.002	<0.001
	Catholic/Protestant	3895	158 765	**24.5**	18.8	33.5	**1**	‐	‐	‐	
	Animist/Atheist	1736	47 904	**36.2**	30.5	46.2	**1.23**	1.15	1.32	<0.001	
Mother's education level	No education	11 805	425 842	**27.7**	23.5	33.0	**1**	‐	‐	‐	0.184
	Primary	860	36 112	**23.8**	19.5	29.0	**0.97**	0.91	1.04	0.445	
	Post primary	175	8751	**20.0**	17.4	22.7	**0.88**	0.76	1.02	0.094	
Mother's marital status	Single/Widow/Divorced	252	9505	**26.5**	20.6	34.6	**1.13**	1.00	1.29	0.053	0.107
	Monogamous union	6644	251 172	**26.5**	22.0	32.1	**1**	‐	‐	‐	
	Polygamous union	5944	209 916	**28.3**	24.0	33.9	**1.02**	0.99	1.06	0.239	
Mother's residence duration	<3 years	388	12 312	**31.5**	25.2	39.3	**1**	‐	‐	‐	<0.001
	3 years or more	12 453	458 399	**27.2**	22.9	32.5	**0.71**	0.64	0.79	<0.001	
Child's birth order (live births)	1	2665	87 220	**30.6**	25.4	37.0	**1.25**	1.19	1.31	<0.001	<0.001
	2–3	3853	157 971	**24.4**	20.4	29.5	**1**	‐	‐	‐	
	4–5	2940	117 883	**24.9**	21.1	29.7	**1.02**	0.97	1.07	0.470	
	6–17	3383	107 636	**31.4**	27.2	36.8	**1.23**	1.17	1.29	<0.001	
Child's birth interval length from previous live birth (months)	First birth	2665	87 220	**30.6**	25.4	37.0	**0.85**	0.81	0.90	<0.001	<0.001
<24	2576	65 212	**39.5**	35.2	44.9	**1**	‐	‐	‐	
24–35	4461	161 634	**27.6**	23.7	32.6	**0.73**	0.70	0.77	<0.001	
36–48	2153	99 556	**21.6**	18.5	25.4	**0.62**	0.59	0.66	<0.001	
≥48	986	57 089	**17.3**	14.5	20.6	**0.54**	0.50	0.58	<0.001	
Child's birth interval length to next live birth (months)	<24	3780	51 353	**73.6**	65.4	82.8	**1**	‐	‐	‐	<0.001
24–35	3871	149 784	**25.8**	22.8	29.6	**0.36**	0.34	0.38	<0.001	
	36–47	1291	89 763	**14.4**	11.8	17.6	**0.21**	0.20	0.23	<0.001	
	≥48	390	37 993	**10.3**	8.3	12.6	**0.16**	0.15	0.18	<0.001	
	Last birth	3509	141 819	**24.7**	20.2	30.4	**0.37**	0.35	0.39	<0.001	
Child's sex	Boy	6812	239 389	**28.5**	24.0	34.0	**1**	‐	‐	‐	<0.001
	Girl	6028	231 309	**26.1**	22.0	31.1	**0.91**	0.88	0.95	<0.001	
Child's age (months)	<1	2612	9108	**286.8**	245.1	337.1	**1**	‐	‐	‐	<0.001
	1–5	1829	44 740	**40.9**	34.8	48.5	**0.14**	0.13	0.15	<0.001	
	6–11	1561	52 089	**30.0**	23.1	39.8	**0.10**	0.10	0.11	<0.001	
	12–17	2397	50 089	**47.9**	38.7	60.1	**0.17**	0.16	0.18	<0.001	
	18–23	468	49 144	**9.5**	7.4	12.5	**0.03**	0.03	0.04	<0.001	
	24–35	2256	93 900	**24.0**	19.0	30.9	**0.09**	0.08	0.09	<0.001	
	36–59	1718	171 643	**10.0**	8.2	12.4	**0.04**	0.03	0.04	<0.001	

a Data not shown to comply with the ethical requirement of the Burkina Faso MoH.

While 25% of children lived within 2 km of a facility, 21% between 2 and 4 km, 24% between 4 and 7 km and 30% lived beyond 7 km. Children (excluding Kantchari cluster) were further away from a hospital: 24% of children lived within 10 km of a hospital, 61% between 10 and 20 km and 15% beyond 20 km. On average, children lived 5 km away from a facility (up to 23 km) and 14 km away from a hospital (up to 37 km). For the vast majority of children, the closest facility was a primary health facility (96%). Associations of covariates with distance to care are shown in Table S7a–c).

After controlling for forced variables and potential confounding factors, there was some evidence for an increasing trend in neonatal child mortality with increasing distance to care (*P* = 0.014), but not for post‐neonatal under‐five child mortality (*P* = 0.488) (Table 3). Neonates who lived 7 km or more from a facility had a 19% higher mortality rate compared to neonates who lived within 2 km of a facility (RR = 1.19; 95%CI 1.03, 1.38; *P* = 0.021). By contrast, in the post‐neonatal period (1 to 59 months), children who lived 7 km or more from a facility had only a 4% higher mortality rate compared to children who lived within 2 km of a facility (RR = 1.04; 95%CI 0.95, 1.13; *P* = 0.392). Subdividing the neonatal period, evidence for an effect was observed in the early neonatal period (*P* = 0.028) but not in the late neonatal period although rate ratios were consistent with a trend (*P* = 0.479). In the first week of life, the mortality rate was 18% higher for neonates who lived 7 km or more from a facility than for neonates who lived 2 km of a facility (RR = 1.18; 95%CI 1.00, 1.39; *P* = 0.056). There was no statistical evidence for an increasing trend in either neonatal mortality (*P* = 0.701) or post‐neonatal child mortality (*P* = 0.162) with increasing distance to the closest hospital.

**Table 3:** Association of distance to the closest health facility or hospital with child mortality

		Number of deaths	Person‐years	Rate per 1000 person‐years	95% CI		Cluster‐adjusted analysis					Cluster‐adjusted multivariable analysis^a^				
							Rate ratio	95% CI		*P*	Likelihood ratio test *P* value	Rate ratio	95% CI		*P*	Likelihood ratio test *P* value
**Early neonatal mortality**																
Distance to the closest facility (km)	<2	378	529	**714.9**	569.0	905.8	**1**	‐	‐	‐	0.001	**1**	‐	‐	‐	0.028
2–4	302	450	**671.5**	498.5	907.0	**0.93**	0.79	1.10	0.407		**0.91**	0.77	1.07	0.252	
	4–7	439	513	**856.2**	701.3	1057.5	**1.14**	0.97	1.33	0.109		**1.06**	0.90	1.23	0.492	
	>7	613	655	**936.6**	779.4	1114.9	**1.30**	1.10	1.54	0.002		**1.18**	1.00	1.39	0.056	
Distance to the closest hospital (excluding Kantchari cluster) (km)	<10	341	460	**741.6**	501.5	1136.6	**1**	‐	‐	‐	0.198	**1**	‐	‐	‐	0.280
10–20	984	1180	**833.7**	687.7	1013.6	**1.14**	0.99	1.31	0.074		**1.12**	0.97	1.28	0.118	
> 20	259	304	**852.0**	738.3	975.1	**1.11**	0.91	1.36	0.309		**1.07**	0.88	1.30	0.507	
**Late neonatal mortality**																
Distance to the closest facility (km)	< 2	180	1724	**104.4**	83.0	133.0	**1**	‐	‐	‐	0.161	**1**	‐	‐	‐	0.479
2–4	164	1466	**111.9**	84.2	150.5	**1.02**	0.81	1.28	0.883		**1.03**	0.81	1.30	0.810	
4–7	223	1668	**133.7**	108.1	168.6	**1.22**	0.97	1.54	0.091		**1.16**	0.92	1.47	0.208	
>7	313	2127	**147.2**	125.7	174.0	**1.26**	0.98	1.62	0.070		**1.18**	0.92	1.52	0.202	
Distance to the closest hospital (excluding Kantchari cluster) (km)	<10	178	1497	**118.9**	97.7	145.0	**1**	‐	‐	‐	0.126	**1**	‐	‐	‐	0.150
10–20	475	3843	**123.6**	100.4	155.6	**0.91**	0.74	1.11	0.364		**0.89**	0.72	1.09	0.249	
>20	142	988	**143.7**	106.2	192.1	**1.17**	0.87	1.56	0.298		**1.11**	0.83	1.49	0.492	
**Neonatal mortality**																
Distance to the closest facility (km)	<2	558	2249	**248.1**	201.1	308.2	**1**	‐	‐	‐	<0.001	**1**	‐	‐	‐	0.014
2–4	466	1912	**243.8**	183.3	325.6	**0.96**	0.83	1.10	0.529		**0.94**	0.82	1.08	0.397	
4–7	662	2176	**304.2**	263.1	354.9	**1.16**	1.02	1.33	0.029		**1.09**	0.95	1.25	0.219	
>7	926	2775	**333.7**	289.1	383.0	**1.30**	1.12	1.51	0.001		**1.19**	1.03	1.38	0.021	
Distance to the closest hospital (excluding Kantchari cluster) (km)	<10	519	1953	**265.7**	193.4	372.3	**1**	‐	‐	‐	0.431	**1**	‐	‐	‐	0.701
10–20	1459	5013	**291.1**	245.7	346.9	**1.06**	0.94	1.19	0.378		**1.03**	0.92	1.17	0.575	
>20	401	1289	**311.0**	260.5	366.2	**1.12**	0.94	1.34	0.200		**1.08**	0.90	1.28	0.404	
**Post‐neonatal under‐five mortality**																
Distance to the closest facility (km)	<2	2069	117 394	**17.6**	14.8	20.9	**1**	‐	‐	‐	0.094	**1**	‐	‐	‐	0.488
2–4	1824	98 602	**18.5**	14.5	23.5	**1.01**	0.93	1.09	0.776		**0.97**	0.90	1.05	0.491	
4–7	2524	110 411	**22.9**	19.2	27.8	**1.07**	0.98	1.16	0.127		**1.00**	0.92	1.08	0.977	
>7	3812	135 193	**28.2**	23.2	34.8	**1.11**	1.02	1.22	0.021		**1.04**	0.95	1.13	0.392	
Distance to the closest hospital (excluding Kantchari cluster) (km)	<10	1799	102 460	**17.6**	13.7	22.6	**1**	‐	‐	‐	0.040	**1**	‐	‐	‐	0.162
10–20	5608	255 144	**22.0**	17.6	28.0	**1.09**	1.02	1.17	0.012		**1.07**	1.00	1.14	0.059	
>20	1606	63 911	**25.1**	21.2	30.7	**1.09**	0.98	1.21	0.114		**1.04**	0.94	1.16	0.391	
**Under‐five child mortality**																
Distance to the closest facility (km)	<2	2627	119 643	**22.0**	18.5	25.9	**1**	‐	‐	‐	0.002	**1**	‐	‐	‐	0.051
2–4	2290	100 513	**22.8**	17.9	28.9	**1.00**	0.93	1.07	0.958		**0.96**	0.90	1.03	0.274	
4–7	3186	112 587	**28.3**	24.2	33.7	**1.09**	1.01	1.17	0.027		**1.02**	0.95	1.09	0.642	
>7	4738	137 968	**34.3**	28.9	41.3	**1.15**	1.06	1.25	0.001		**1.07**	0.99	1.16	0.088	
Distance to the closest hospital (excluding Kantchari cluster) (km)	<10	2318	104 413	**22.2**	17.1	28.9	**1**	‐	‐	‐	0.029	**1**	‐	‐	‐	0.181
10–20	7067	260 157	**27.2**	22.0	34.0	**1.09**	1.02	1.16	0.009		**1.06**	1.00	1.12	0.065	
>20	2007	65 200	**30.8**	26.8	36.2	**1.10**	0.99	1.21	0.065		**1.05**	0.96	1.14	0.326	

a Forced variables (household's wealth quintile, mother's age at birth, child's gender and age) and potential confounders included (mother's ethnicity, religion, education level, residence duration, marital status, child's birth order, birth intervals).

With respect to self‐reported care seeking behaviours, there was strong evidence for a decreasing trend in care seeking with increasing distance to care after adjusting for forced variables and potential confounding factors (*P* ≤ 0.005) (Table 4). For instance, women who lived 7 km or more from a facility had 78% lower odds of facility delivery (OR = 0.22; 95%CI 0.15, 0.33; *P* < 0.001) than women who lived within 2 km of a facility.

**Table 4:** Association of distance to the closest health facility with self‐reported care seeking behaviours

	Distance to the closest facility (km)	Total	%	95% CI		Cluster‐adjusted analysis					Cluster‐adjusted multivariable analysis^a^				
						Odds ratio	95% CI		*P*	Likelihood ratio test *P* value	Odds ratio	95% CI		*P*	Likelihood ratio test *P* value
Modern contraception use	<2	1388	**34.7**	28.0	41.9	**1**	–	–	–	<0.001	**1**	–	–	–	<0.001
2–4	1069	**26.7**	20.8	33.4	**0.73**	0.60	0.89	0.002		**0.80**	0.65	0.98	0.033	
4–7	1189	**26.8**	20.3	34.5	**0.62**	0.51	0.77	<0.001		**0.68**	0.55	0.84	<0.001	
>7	1464	**19.1**	11.8	29.6	**0.54**	0.43	0.68	<0.001		**0.58**	0.46	0.75	<0.001	
Four or more ANC visits in a facility	<2	1535	**58.8**	51.7	65.6	**1**	–	–	–	<0.001	**1**	–	–	–	<0.001
2–4	1170	**53.5**	45.0	61.8	**0.80**	0.66	0.96	0.016		**0.85**	0.70	1.02	0.083	
4–7	1299	**49.3**	41.5	57.2	**0.65**	0.53	0.79	<0.001		**0.68**	0.55	0.83	<0.001	
>7	1653	**35.8**	25.2	47.9	**0.49**	0.39	0.61	<0.001		**0.51**	0.41	0.64	<0.001	
Facility delivery	<2	1535	**93.5**	89.2	96.1	**1**	–	–	–	<0.001	**1**	–	–	–	<0.001
2–4	1170	**89.1**	83.0	93.2	**0.59**	0.42	0.83	0.003		**0.64**	0.45	0.91	0.012	
4–7	1299	**81.4**	69.0	89.5	**0.35**	0.25	0.49	<0.001		**0.38**	0.27	0.54	<0.001	
>7	1653	**59.3**	38.5	77.2	**0.21**	0.15	0.31	<0.001		**0.22**	0.15	0.33	<0.001	
Care seeking for childhood illness in a facility	<2	484	**59.3**	52.1	66.1	**1**	–	–	–	0.001	**1**	–	–	–	0.005
2–4	375	**53.3**	43.2	63.2	**0.80**	0.60	1.07	0.138		**0.84**	0.62	1.14	0.269	
4–7	445	**50.3**	40.7	59.9	**0.72**	0.53	0.96	0.025		**0.76**	0.55	1.04	0.081	
>7	533	**41.7**	26.2	58.9	**0.51**	0.36	0.71	<0.001		**0.52**	0.36	0.74	<0.001	

a Forced variables (household's wealth quintile, mother's age at interview, child's gender and age) and potential confounders (mother's ethnicity, religion, education level, residence duration, marital status, parity).

There was no evidence that the associations of distance to the closest facility or hospital with neonatal or post‐neonatal mortality varied by household wealth tertile (*P* > 0.470) or mother's school attendance (*P* > 0.101). Similar findings were found with respect to the associations of distance to care with care seeking behaviours.

### Sensitivity analyses

Facilities are often located in relatively larger villages where more commodities (e.g. market, school) are available compared with more remote villages. Therefore, living further away from a village with a facility might lead to greater poverty, lower education level, or, because of lower contraception uptake, shorter birth interval lengths. Excluding household wealth quintile, mother's level of education, and birth intervals in turn had no important effect on the final results, although excluding birth intervals slightly increased the strength of the association of distance to care with neonatal and post‐neonatal under‐five child mortality (Table S8). Similarly, excluding household wealth quintile or mother's education level from the multivariable analysis of care seeking behaviours made no or little difference to the results (Table S9).

## Discussion

In our study population, around a third of women and their children lived 7 km or more from a facility. We observed a steep decline in self‐reported facility delivery and other care seeking behaviours with increasing distance to care. However, while there was some evidence for an increase in the neonatal mortality rate with increasing distance to care (*P* = 0.014), no evidence was found for an increase in the post‐neonatal under‐five child mortality rate with increasing distance (*P* = 0.488). Furthermore, in the neonatal period, evidence for an effect of distance to care on mortality was observed in the early neonatal period (*P* = 0.028), but not in the late neonatal period although rate ratios were consistent with a trend (*P* = 0.479). A meta‐analysis of 13 studies conducted in LMIC also reported stronger effects of distance to care on perinatal and neonatal mortality than on infant and under‐five child mortality 12 and analysis of 29 Demographic and Health Surveys reported similar findings 11.

In our study, 66% of neonatal deaths occurred in the first week after birth, with over half of these deaths occurring within 2 days of birth. The greater effect of distance to care in the early neonatal period (0 to 6 days) compared with the late neonatal (7 to 27 days) and post‐neonatal (1 to 59 months) periods seems plausible given that neonates are more vulnerable to rapidly progressive conditions, so that distance to care is an important determinant of early neonatal survival. In the late neonatal period, it is noteworthy, however, that rate ratios were similar to or larger than estimates in early neonatal mortality. Thus, the lack of evidence may reflect the lower power due to fewer deaths rather than a weaker association.

Furthermore, suboptimal quality of childbirth care and management of key conditions including malaria, pneumonia and diarrhoea might have weakened the strength of the associations of distance to care with neonatal and post‐neonatal mortality we observed. In Malawi, proximity to delivery care was also strongly associated with higher facility delivery, but not with lower early neonatal mortality 8. In contrast, in Ethiopia, evidence that proximity to comprehensive EmONC that was associated with lower early neonatal mortality was observed 20. In our study, a relatively high neonatal mortality rate (20.3 per 1000 live births) was found even in neonates who lived within 2 km of a facility and despite the fact that 94% of their mothers reported having given birth in a facility. Similarly, although about 60% of women living within 2 km of a facility reported seeking care for their child, post‐neonatal under‐five child mortality within this distance was still high (79.6 per 1000 children). In the 2014 SARA, three of the seven basic EmONC signal functions had poor availability, and only 61%, 41% and 63% of staff were trained in EmONC, neonatal resuscitation and IMCI guidelines respectively (Table 1). In 2011, while 91% of children with presumed uncomplicated malaria were observed to receive an ACT, only 34% of children with signs of pneumonia and 30% of children with diarrhoea were correctly prescribed antibiotics and ORS respectively 21. The very low proportion of women who reported having given birth by caesarean section for their last delivery (only 1% overall), well below the expected 5% to 10% rate 22, also suggests issues in the quality of childbirth care.

Lastly, in some contexts, the strength of the association of distance to care with child mortality could be weakened if there was a large coverage of community case management of childhood illnesses. However, we do not believe that this has affected our findings substantially. At the time of our survey, the proportion of community health worker (CHW) in surveyed villages with ACT and ORS available was 43.6% and 28.0%, respectively, and only 11.5% of mothers reported to have visited a CHW for treating their children (fever, diarrhoea or cough/difficult breathing). Another survey conducted in Burkina Faso during 2011‐2013 reported that between 1% and 9% of sick children consulted a CHW 15.

### Limitations

Our study has some limitations. First, Euclidian distances from each household to the closest facility were calculated. In Ethiopia, an association of under‐five child mortality was observed with travelled distance or walking travel time to care, but not with distance measured as straight line 12. Thus, the use of Euclidian distance rather than network distance may have attenuated the observed associations of distance to care with mortality.

Second, our study excluded communities living in and within 5 km of towns and in villages above 5000 inhabitants and is therefore not representative of the rural population of Burkina Faso. Nevertheless, the 2014 Burkina Faso MoH statistics reported that 40% of the whole population lived 5 km or more from a facility compared with 45% in our study 23. We did not find evidence for an increase in either neonatal mortality (*P* = 0.701) or post‐neonatal under‐five child mortality (*P* = 0.162) with increasing distance to hospital. An obvious explanation for this finding is the infrequency of care seeking in a hospital for both childbirth and childhood illnesses observed in our study (6% or less). Hospitals being located in towns, our study population therefore excluded communities living <5 km away from a hospital. A review has highlighted the fact that some studies suggest that use of hospitals for delivery care only increases substantially when women live very close to a hospital 24. Thus we cannot exclude the possibility that short distances to hospital (<5 km) are associated with increased child survival in Burkina Faso.

Third, distance to care was measured once, at the time of interview. According to Burkina Faso MoH reports, 50 public facilities were constructed between 2010 and 2014 in the health districts containing clusters included in this survey. Although nearly all women (97%) in our study reported having lived for 3 years or more in their village, some children were likely not exposed to the same distance to care throughout the whole study period which may have weakened the observed associations of distance with mortality.

Fourth, mortality data, collected by interviewing women about their pregnancy histories, are subject to errors. Women may be reluctant to report sad events, such as neonatal deaths in particular. In addition, misclassification of very early neonatal deaths as stillbirths may be more likely in remote villages where home‐based deliveries, during which brief signs of life in a newborn may be unnoticed, are more common 25. In our study, home‐based deliveries were reported by 41% of women who lived 7 km or more from a facility in our study compared with 6% of women within 2 km of a facility. In 2015, 30% of under‐five deaths were estimated to occur in the neonatal period in Burkina Faso 26 compared to 20% in our study. We cannot exclude the possibility of some under‐reporting of neonatal deaths, and if women in more remote villages were more likely to under‐report neonatal deaths, this would have weakened the observed association of distance with neonatal mortality.

Fifth, we cannot exclude possible residual confounding. In our study, we found evidence for decreasing trends in vitamin A uptake in the past 6 months and in sanitation related behaviours with increasing distance to care. These risk factors for mortality, which could not be adjusted for in our analyses (only collected during interviews on behaviours), could have resulted in an overestimate of the association between distance and mortality.

## Conclusion

In summary, despite strong evidence for an association of distance to facility with care seeking behaviours, there was only some evidence for an effect of distance to care on neonatal mortality, particularly in the first week of life, and no evidence for an effect of distance on post‐neonatal under‐five child mortality.

In 2016, free care for under‐five children was introduced in Burkina Faso, which removes part of the financial barrier to care seeking. Our findings suggest that better geographic access to facility can promote care seeking behaviours, but also suggest that improving geographic access alone without improving quality of care may have limited impact on child mortality.

## Supporting information


**Table S1.** Cluster‐adjusted associations with neonatal mortality.
**Table S2.** Cluster‐adjusted associations with post‐neonatal under‐five child mortality.
**Table S3.** Cluster‐adjusted associations with modern contraception use.
**Table S4.** Cluster‐adjusted associations with four or more ANC visits in a health facility.
**Table S5.** Cluster‐adjusted associations with delivery in a health facility.
**Table S6.** Cluster‐adjusted associations with care seeking in a health facility for childhood illness.
**Table S7.** (a) Distribution of distance to the closest facility in under‐five children at risk during the study period. (b) Distribution of distance to the closest hospital in under‐five children at risk during the study period. (c) Distribution of distance to the closest facility in mothers interviewed about their family behaviours.
**Table S8.** Sensitivity analysis for the association of distance to the closest facility or hospital with child mortality.
**Table S9.** Sensitivity analysis for the association of distance to the closest facility with care seeking behaviours.
