# Supplementary material for: Distance to care, care seeking and child mortality in rural Burkina Faso: findings from a population‐based cross‐sectional survey
Source: Trop Med Int Health. 2018 Nov 18;24(1):31–42. doi: 10.1111/tmi.13170 (PMC6378618; doi:10.1111/tmi.13170)
Supplement: Supplementary file 1 — Table S1. Cluster‐adjusted associations with neonatal mortality. Table S2. Cluster‐adjusted associations with post‐neonatal under‐five child mortality. Table S3. Cluster‐adjusted associations with modern contraception use. Table S4. Cluster‐adjusted associations with four or more ANC visits in a health facility. Table S5. Cluster‐adjusted associations with delivery in a health facility. Table S6. Cluster‐adjusted associations with care seeking in a health facility for childhood illness. Table S7. (a) Distribution of distance to the closest facility in under‐five children at risk during the study period. (b) Distribution of distance to the closest hospital in under‐five children at risk during the study period. (c) Distribution of distance to the closest facility in mothers interviewed about their family behaviours. Table S8. Sensitivity analysis for the association of distance to the closest facility or hospital with child mortality. Table S9. Sensitivity analysis for the association of distance to the closest facility with care seeking behaviours. [file TMI-24-31-s001.docx]

Supplementary material, table 1: Cluster-adjusted associations with neonatal mortality

|  |  | Number of deaths | Person-years | Rate per 1,000 pyrs | 95% CI | | Cluster-adjusted analysis | | | | |
| --- | --- | --- | --- | --- | --- | --- | --- | --- | --- | --- | --- |
|  |  |  |  |  |  |  | Rate Ratio | 95% CI | | p | Likelihood ratio test p value |
|  |  |  |  |  |  |  |  |  |  |  |  |
|  |  |  |  |  |  |  |  |  |  |  |  |
| Household wealth quintile | Poorest | 451 | 1,481 | **304.6** | 236.0 | 396.3 | **1** | - | - | - | 0.185 |
|  | 2nd quintile | 511 | 1,620 | **315.4** | 254.2 | 394.6 | **1.04** | 0.92 | 1.18 | 0.534 |  |
|  | 3rd quintile | 506 | 1,758 | **287.8** | 233.2 | 359.0 | **0.96** | 0.84 | 1.09 | 0.496 |  |
|  | 4th quintile | 519 | 1,913 | **271.3** | 229.5 | 322.2 | **0.92** | 0.81 | 1.04 | 0.191 |  |
|  | Least poor | 598 | 2,228 | **268.4** | 232.0 | 311.5 | **0.91** | 0.80 | 1.04 | 0.159 |  |
| Mother's age at birth (years) | 14 - 20 | 789 | 1,994 | **395.6** | 336.5 | 467.8 | **1.73** | 1.54 | 1.94 | < 0.001 | < 0.001 |
|  | 21 - 25 | 557 | 2,473 | **225.2** | 188.2 | 271.1 | **0.99** | 0.87 | 1.11 | 0.815 |  |
|  | 26 - 30 | 464 | 2,024 | **229.2** | 187.4 | 282.8 | **1** | - | - | - |  |
|  | 31 - 35 | 398 | 1,480 | **268.9** | 223.9 | 325.7 | **1.18** | 1.03 | 1.35 | 0.016 |  |
|  | 36 - 49 | 402 | 1,136 | **353.9** | 310.2 | 405.9 | **1.54** | 1.35 | 1.77 | < 0.001 |  |
| Mother's ethnicity* |  |  |  |  |  |  |  |  |  |  | < 0.001 |
| Mother's religion | Muslim | 1,475 | 5,140 | **287.0** | 234.7 | 354.7 | **1.07** | 0.96 | 1.19 | 0.205 | 0.001 |
|  | Catholic/Protestant | 768 | 3,026 | **253.8** | 219.2 | 301.2 | **1** | - | - | - |  |
|  | Animist/Atheist | 368 | 944 | **389.7** | 287.9 | 522.4 | **1.34** | 1.16 | 1.55 | < 0.001 |  |
| Mother's education level | No education | 2,340 | 8,096 | **289.0** | 245.3 | 342.6 | **1** | - | - | - | 0.301 |
|  | Primary | 221 | 779 | **283.8** | 242.6 | 332.0 | **1.02** | 0.89 | 1.17 | 0.780 |  |
|  | Post primary | 50 | 236 | **211.5** | 162.7 | 277.6 | **0.81** | 0.61 | 1.08 | 0.145 |  |
| Mother's marital status | Single/Widow/Divorced | 57 | 176 | **324.7** | 218.6 | 527.4 | **1.11** | 0.85 | 1.45 | 0.432 | 0.498 |
|  | Monogamous union | 1,456 | 5,134 | **283.6** | 243.1 | 331.9 | **1** | - | - | - |  |
|  | Polygamous union | 1,099 | 3,799 | **289.3** | 242.6 | 349.4 | **0.97** | 0.89 | 1.05 | 0.432 |  |
| Mother's residence duration | Less than 3 years | 127 | 346 | **366.9** | 286.6 | 472.5 | **1** | - | - | - | 0.001 |
|  | 3 years or more | 2,485 | 8,765 | **283.5** | 242.3 | 333.4 | **0.72** | 0.60 | 0.87 | < 0.001 |  |
| Child's birth order | 1 | 703 | 1,644 | **427.7** | 360.5 | 510.0 | **1.96** | 1.76 | 2.17 | < 0.001 | < 0.001 |
| (live births) | 2 - 3 | 663 | 3,012 | **220.2** | 180.8 | 269.8 | **1** | - | - | - |  |
|  | 4 - 5 | 524 | 2,265 | **231.3** | 191.8 | 281.1 | **1.04** | 0.93 | 1.17 | 0.477 |  |
|  | 6 - 17 | 722 | 2,191 | **329.6** | 287.5 | 379.7 | **1.45** | 1.30 | 1.61 | < 0.001 |  |
| Child's birth interval length from previous live birth (months) | First birth | 703 | 1,644 | **427.7** | 360.5 | 510.0 | **1.00** | 0.89 | 1.12 | 0.991 | < 0.001 |
|  | < 24 | 519 | 1,152 | **450.6** | 380.9 | 536.6 | **1** | - | - | - |  |
|  | 24 - 35 | 772 | 3,141 | **245.8** | 216.7 | 279.4 | **0.56** | 0.50 | 0.63 | < 0.001 |  |
|  | 36 - 47 | 381 | 1,982 | **192.2** | 152.3 | 245.6 | **0.45** | 0.39 | 0.51 | < 0.001 |  |
|  | ≥ 48 | 237 | 1,193 | **198.7** | 157.5 | 252.7 | **0.47** | 0.40 | 0.55 | < 0.001 |  |
| Child's birth interval length to next live birth (months) | < 24 | 941 | 700 | **1344.9** | 1082.5 | 1658.1 | **1** | - | - | - | < 0.001 |
|  | 24 - 35 | 454 | 1,726 | **263.0** | 217.8 | 318.5 | **0.19** | 0.17 | 0.22 | < 0.001 |  |
|  | 36 - 47 | 112 | 795 | **140.9** | 107.1 | 187.7 | **0.10** | 0.09 | 0.13 | < 0.001 |  |
|  | ≥ 48 | 23 | 155 | **148.4** | 82.5 | 295.3 | **0.11** | 0.07 | 0.17 | < 0.001 |  |
|  | Last birth | 1,082 | 5,736 | **188.6** | 156.9 | 228.2 | **0.14** | 0.13 | 0.15 | < 0.001 |  |
| Child's sex | Boy | 1,505 | 4,647 | **323.9** | 274.4 | 384.8 | **1** | - | - | - | < 0.001 |
|  | Girl | 1,106 | 4,465 | **247.7** | 211.0 | 291.9 | **0.76** | 0.71 | 0.82 | < 0.001 |  |
| * Data not shown to comply with the ethical requirement of the Burkina Faso MoH | | | | | | | |  |  |  |  |

Supplementary material, table 2: Cluster-adjusted associations with post-neonatal under-five child mortality

|  |  | Number of deaths | Person-years | Rate per 1,000 pyrs | 95% CI | | Cluster-adjusted analysis | | | | |
| --- | --- | --- | --- | --- | --- | --- | --- | --- | --- | --- | --- |
|  |  |  |  |  |  |  | Rate Ratio | 95% CI | | p | Likelihood ratio test p value |
|  |  |  |  |  |  |  |  |  |  |  |  |
|  |  |  |  |  |  |  |  |  |  |  |  |
| Household wealth quintile | Poorest | 1,819 | 69,066 | **26.3** | 21.6 | 32.5 | **1** | - | - | - | < 0.001 |
|  | 2nd quintile | 1,916 | 79,164 | **24.2** | 19.8 | 29.9 | **0.96** | 0.90 | 1.02 | 0.175 |  |
|  | 3rd quintile | 1,963 | 89,817 | **21.9** | 17.9 | 27.0 | **0.87** | 0.82 | 0.93 | < 0.001 |  |
|  | 4th quintile | 2,045 | 99,756 | **20.5** | 17.2 | 24.6 | **0.85** | 0.80 | 0.91 | < 0.001 |  |
|  | Least poor | 2,380 | 118,243 | **20.1** | 16.4 | 24.8 | **0.83** | 0.78 | 0.89 | < 0.001 |  |
| Mother's age at birth (years) | 14 - 20 | 2,427 | 101,552 | **23.9** | 19.9 | 29.2 | **1.13** | 1.07 | 1.20 | < 0.001 | < 0.001 |
|  | 21 - 25 | 2,681 | 128,641 | **20.8** | 17.3 | 25.4 | **1.00** | 0.95 | 1.06 | 0.895 |  |
|  | 26 - 30 | 2,165 | 105,187 | **20.6** | 16.9 | 25.3 | **1** | - | - | - |  |
|  | 31 - 35 | 1,657 | 73,552 | **22.5** | 19.0 | 26.9 | **1.11** | 1.04 | 1.18 | 0.002 |  |
|  | 36 - 49 | 1,295 | 52,464 | **24.7** | 20.5 | 29.9 | **1.23** | 1.15 | 1.32 | < 0.001 |  |
| Mother's ethnicity* | |  |  |  |  |  |  |  |  |  | < 0.001 |
| Mother's religion | Muslim | 5,734 | 258,884 | **22.1** | 17.8 | 27.9 | **1.09** | 1.03 | 1.15 | 0.003 | < 0.001 |
|  | Catholic/Protestant | 3,127 | 155,739 | **20.1** | 15.0 | 28.4 | **1** | - | - | - |  |
|  | Animist/Atheist | 1,368 | 46,960 | **29.1** | 24.0 | 38.6 | **1.21** | 1.12 | 1.31 | < 0.001 |  |
| Mother's education level | No education | 9,465 | 417,746 | **22.7** | 19.0 | 27.3 | **1** | - | - | - | 0.005 |
|  | Primary | 639 | 35,333 | **18.1** | 14.4 | 22.7 | **0.92** | 0.84 | 0.99 | 0.035 |  |
|  | Post primary | 125 | 8,514 | **14.7** | 12.1 | 17.7 | **0.80** | 0.67 | 0.96 | 0.014 |  |
| Mother's marital status | Single/Widow/Divorced | 195 | 9,329 | **20.9** | 16.1 | 27.2 | **1.16** | 1.00 | 1.34 | 0.047 | 0.001 |
|  | Monogamous union | 5,188 | 246,038 | **21.1** | 17.3 | 26.0 | **1** | - | - | - |  |
|  | Polygamous union | 4,845 | 206,118 | **23.5** | 19.7 | 28.5 | **1.07** | 1.03 | 1.12 | 0.001 |  |
| Mother's residence duration | Less than 3 years | 261 | 11,966 | **21.8** | 17.0 | 28.0 | **1** | - | - | - | 0.001 |
|  | 3 years or more | 9,968 | 449,634 | **22.2** | 18.5 | 26.9 | **0.81** | 0.72 | 0.92 | 0.001 |  |
| Child's birth order | 1 | 1,962 | 85,577 | **22.9** | 18.8 | 28.2 | **1.11** | 1.05 | 1.17 | < 0.001 | < 0.001 |
| (live births) | 2 - 3 | 3,190 | 154,960 | **20.6** | 17.0 | 25.1 | **1** | - | - | - |  |
|  | 4 - 5 | 2,416 | 115,618 | **20.9** | 17.4 | 25.3 | **1.01** | 0.96 | 1.07 | 0.695 |  |
|  | 6 - 17 | 2,661 | 105,446 | **25.2** | 21.6 | 29.9 | **1.16** | 1.11 | 1.23 | < 0.001 |  |
| Child's birth interval length from previous live birth (months) | First birth | 1,962 | 85,577 | **22.9** | 18.8 | 28.2 | **0.79** | 0.74 | 0.84 | < 0.001 | < 0.001 |
|  | < 24 | 2,057 | 64,060 | **32.1** | 28.6 | 36.5 | **1** | - | - | - |  |
|  | 24 - 35 | 3,689 | 158,493 | **23.3** | 19.6 | 28.0 | **0.76** | 0.72 | 0.81 | < 0.001 |  |
|  | 36 - 47 | 1,772 | 97,574 | **18.2** | 15.5 | 21.4 | **0.65** | 0.61 | 0.69 | < 0.001 |  |
|  | ≥ 48 | 749 | 55,897 | **13.4** | 11.2 | 16.1 | **0.53** | 0.48 | 0.57 | < 0.001 |  |
| Child's birth interval length to next live birth (months) | < 24 | 2,839 | 50,653 | **56.0** | 50.5 | 62.4 | **1** | - | - | - | < 0.001 |
|  | 24 - 35 | 3,417 | 148,058 | **23.1** | 20.1 | 26.8 | **0.43** | 0.41 | 0.45 | < 0.001 |  |
|  | 36 - 47 | 1,179 | 88,968 | **13.3** | 10.8 | 16.3 | **0.26** | 0.24 | 0.28 | < 0.001 |  |
|  | ≥ 48 | 367 | 37,839 | **9.7** | 7.8 | 11.9 | **0.21** | 0.19 | 0.23 | < 0.001 |  |
|  | Last birth | 2,427 | 136,083 | **17.8** | 14.4 | 22.2 | **0.36** | 0.34 | 0.38 | < 0.001 |  |
| Child's sex | Boy | 5,307 | 234,743 | **22.6** | 18.8 | 27.5 | **1** | - | - | - | 0.025 |
|  | Girl | 4,922 | 226,845 | **21.7** | 18.1 | 26.2 | **0.96** | 0.92 | 0.99 | 0.025 |  |
| Child's age (months) | 1-5 | 1,829 | 44,736 | **40.9** | 34.8 | 48.5 | **1** | - | - | - | < 0.001 |
|  | 6 - 11 | 1,561 | 52,089 | **30.0** | 23.1 | 39.8 | **0.74** | 0.69 | 0.79 | < 0.001 |  |
|  | 12 - 17 | 2,397 | 50,089 | **47.9** | 38.7 | 60.1 | **1.18** | 1.11 | 1.26 | < 0.001 |  |
|  | 18 - 23 | 468 | 49,144 | **9.5** | 7.4 | 12.5 | **0.24** | 0.21 | 0.26 | < 0.001 |  |
|  | 24 - 35 | 2,256 | 93,900 | **24.0** | 19.0 | 30.9 | **0.60** | 0.56 | 0.64 | < 0.001 |  |
|  | 36 - 59 | 1,718 | 171,643 | **10.0** | 8.2 | 12.4 | **0.25** | 0.24 | 0.27 | < 0.001 |  |
| * Data not shown to comply with the ethical requirement of the Burkina Faso MoH | | | | | | | |  |  |  |  |

Supplementary material, table 3: Cluster-adjusted associations with modern contraception use

|  |  | Total | Modern contra-ception use (%) | 95% CI | | Cluster-adjusted analysis | | | | |
| --- | --- | --- | --- | --- | --- | --- | --- | --- | --- | --- |
|  |  |  |  |  |  | Odds Ratio | 95% CI | | p | Likelihood ratio test p value |
|  |  |  |  |  |  |  |  |  |  |  |
|  |  |  |  |  |  |  |  |  |  |  |
| Household wealth quintile | Poorest | 789 | **21.8** | 14.1 | 32.1 | **1** | - | - | - | <0.001 |
|  | 2nd quintile | 922 | **23.0** | 16.2 | 31.6 | **1.05** | 0.83 | 1.34 | 0.669 |  |
|  | 3rd quintile | 986 | **26.0** | 20.2 | 32.7 | **1.21** | 0.95 | 1.53 | 0.115 |  |
|  | 4th quintile | 1,091 | **27.2** | 21.0 | 34.5 | **1.28** | 1.02 | 1.62 | 0.036 |  |
|  | Least poor | 1,280 | **32.3** | 24.9 | 40.8 | **1.66** | 1.33 | 2.09 | <0.001 |  |
| Mother's age at interview (years) | 14-20 | 755 | **20.0** | 13.3 | 28.9 | **1** | - | - | - | <0.001 |
|  | 21-25 | 1,413 | **24.9** | 18.4 | 32.8 | **1.35** | 1.08 | 1.69 | 0.009 |  |
|  | 26-30 | 1,203 | **28.9** | 22.2 | 36.7 | **1.70** | 1.35 | 2.14 | <0.001 |  |
|  | 31-35 | 834 | **31.3** | 24.0 | 39.7 | **1.86** | 1.46 | 2.38 | <0.001 |  |
|  | 36-49 | 853 | **27.9** | 21.9 | 34.8 | **1.64** | 1.28 | 2.10 | <0.001 |  |
| Mother's ethnicity* | |  |  |  |  |  |  |  |  | <0.001 |
| Mother's religion | Muslim | 2,839 | **23.4** | 16.6 | 31.8 | **0.73** | 0.62 | 0.87 | <0.001 | 0.001 |
|  | Catholic/Protestant | 1,803 | **32.7** | 25.9 | 40.4 | **1** | - | - | - |  |
|  | Animist/Atheist | 419 | **23.6** | 14.8 | 35.5 | **0.80** | 0.60 | 1.07 | 0.140 |  |
| Mother's education level | No education | 4,176 | **24.7** | 18.9 | 31.5 | **1** | - | - | - | <0.001 |
|  | Primary | 613 | **36.7** | 28.4 | 45.9 | **1.54** | 1.27 | 1.86 | <0.001 |  |
|  | Post primary | 272 | **35.7** | 27.8 | 44.4 | **1.54** | 1.17 | 2.02 | 0.002 |  |
| Mother's residence duration | Less than 3 years | 320 | **23.1** | 15.0 | 33.9 | **1** | - | - | - | 0.022 |
|  | 3 years or more | 4,741 | **27.0** | 20.7 | 34.3 | **1.38** | 1.04 | 1.83 | 0.025 |  |
| Mother's marital status | Single/Widow/Divorced | 118 | **21.2** | 13.6 | 31.5 | **0.60** | 0.38 | 0.96 | 0.033 | 0.062 |
|  | Monogamous union | 3,083 | **28.1** | 20.9 | 36.5 | **1** | - | - | - |  |
|  | Polygamous union | 1,832 | **24.7** | 19.5 | 30.9 | **0.93** | 0.80 | 1.07 | 0.286 |  |
| Parity | 1 | 896 | **21.8** | 16.2 | 28.6 | **1** | - | - | - | <0.001 |
| (stillbirths and live births) | 2 - 3 | 1,734 | **27.7** | 20.7 | 36.0 | **1.46** | 1.20 | 1.78 | <0.001 |  |
|  | 4 - 5 | 1,278 | **29.5** | 22.4 | 37.7 | **1.65** | 1.34 | 2.04 | <0.001 |  |
|  | 6 - 15 | 1,153 | **26.0** | 19.8 | 33.4 | **1.44** | 1.16 | 1.80 | 0.001 |  |
| * Data not shown to comply with the ethical requirement of the Burkina Faso MoH | | | | | | | |  |  |  |

Supplementary material, table 4: Cluster-adjusted associations with 4 ore more ANC visits in a health facility

|  |  | Total | 4 or more ANC (%) | 95% CI | | Cluster-adjusted analysis | | | | |
| --- | --- | --- | --- | --- | --- | --- | --- | --- | --- | --- |
|  |  |  |  |  |  | Odds Ratio | 95% CI | | p | Likelihood ratio test p value |
|  |  |  |  |  |  |  |  |  |  |  |
|  |  |  |  |  |  |  |  |  |  |  |
| Household wealth quintile | Poorest | 871 | **40.1** | 26.9 | 54.9 | **1** | - | - | - | <0.001 |
|  | 2nd quintile | 1,008 | **44.5** | 34.1 | 55.5 | **1.09** | 0.89 | 1.33 | 0.420 |  |
|  | 3rd quintile | 1,109 | **47.9** | 39.7 | 56.1 | **1.14** | 0.94 | 1.39 | 0.188 |  |
|  | 4th quintile | 1,222 | **51.6** | 44.5 | 58.5 | **1.28** | 1.05 | 1.56 | 0.013 |  |
|  | Least poor | 1,400 | **56.1** | 51.0 | 61.2 | **1.54** | 1.27 | 1.88 | <0.001 |  |
| Mother's age at interview (years) | 14-20 | 811 | **46.5** | 38.0 | 55.2 | **1** | - | - | - | 0.530 |
|  | 21-25 | 1,603 | **47.7** | 39.3 | 56.1 | **1.03** | 0.86 | 1.24 | 0.738 |  |
|  | 26-30 | 1,376 | **48.9** | 40.5 | 57.4 | **1.09** | 0.90 | 1.31 | 0.384 |  |
|  | 31-35 | 914 | **51.5** | 42.4 | 60.5 | **1.18** | 0.96 | 1.45 | 0.116 |  |
|  | 36-49 | 899 | **49.9** | 41.9 | 58.0 | **1.08** | 0.88 | 1.33 | 0.446 |  |
| Mother's ethnicity* | |  |  |  |  |  |  |  |  | 0.003 |
| Mother's religion | Muslim | 3,164 | **44.2** | 33.5 | 55.4 | **0.79** | 0.68 | 0.92 | 0.002 | 0.002 |
|  | Catholic/Protestant | 1,971 | **58.0** | 52.9 | 62.9 | **1** | - | - | - |  |
|  | Animist/Atheist | 471 | **41.6** | 36.0 | 47.4 | **0.72** | 0.56 | 0.93 | 0.010 |  |
| Mother's education level | No education | 4,628 | **47.5** | 38.8 | 56.3 | **1** | - | - | - | 0.031 |
|  | Primary | 680 | **54.9** | 47.4 | 62.1 | **1.22** | 1.02 | 1.45 | 0.031 |  |
|  | Post primary | 298 | **55.7** | 45.5 | 65.5 | **1.25** | 0.97 | 1.61 | 0.083 |  |
| Mother's residence duration | Less than 3 years | 339 | **50.4** | 41.0 | 59.8 | **1** | - | - | - | 0.452 |
|  | 3 years or more | 5,267 | **48.7** | 40.4 | 57.1 | **1.10** | 0.86 | 1.39 | 0.452 |  |
| Mother's marital status | Single/Widow/Divorced | 120 | **41.7** | 33.9 | 49.8 | **0.66** | 0.45 | 0.98 | 0.040 | 0.110 |
|  | Monogamous union | 3,419 | **49.0** | 39.9 | 58.2 | **1** | - | - | - |  |
|  | Polygamous union | 2,038 | **48.9** | 41.0 | 56.9 | **1.01** | 0.89 | 1.14 | 0.865 |  |
| Parity | 1 | 1,020 | **49.2** | 42.6 | 55.8 | **1** | - | - | - | 0.942 |
| (stillbirths and live births) | 2 - 3 | 1,957 | **48.9** | 39.9 | 57.9 | **1.03** | 0.88 | 1.21 | 0.706 |  |
|  | 4 - 5 | 1,408 | **49.6** | 40.5 | 58.7 | **1.05** | 0.88 | 1.25 | 0.562 |  |
|  | 6 - 15 | 1,221 | **47.4** | 38.5 | 56.5 | **1.01** | 0.85 | 1.22 | 0.878 |  |
| * Data not shown to comply with the ethical requirement of the Burkina Faso MoH | | | | | | | |  |  |  |

Supplementary material, table 5: Cluster-adjusted associations with delivery in a health facility

|  |  | Total | Facility delivery (%) | 95% CI | | Cluster-adjusted analysis | | | | |
| --- | --- | --- | --- | --- | --- | --- | --- | --- | --- | --- |
|  |  |  |  |  |  | Odds Ratio | 95% CI | | p | Likelihood ratio test p value |
|  |  |  |  |  |  |  |  |  |  |  |
|  |  |  |  |  |  |  |  |  |  |  |
| Household wealth quintile | Poorest | 871 | **71.8** | 46.5 | 88.2 | **1** | - | - | - | 0.033 |
|  | 2nd quintile | 1,008 | **75.1** | 56.9 | 87.3 | **0.94** | 0.72 | 1.23 | 0.657 |  |
|  | 3rd quintile | 1,109 | **78.6** | 63.6 | 88.6 | **1.02** | 0.78 | 1.33 | 0.903 |  |
|  | 4th quintile | 1,222 | **83.0** | 71.5 | 90.4 | **1.19** | 0.90 | 1.57 | 0.212 |  |
|  | Least poor | 1,400 | **86.5** | 78.5 | 91.8 | **1.38** | 1.04 | 1.82 | 0.025 |  |
| Mother's age at interview (years) | 14-20 | 811 | **82.4** | 66.0 | 91.8 | **1** | - | - | - | <0.001 |
|  | 21-25 | 1,603 | **80.8** | 64.6 | 90.7 | **0.74** | 0.56 | 0.97 | 0.031 |  |
|  | 26-30 | 1,376 | **79.4** | 65.3 | 88.7 | **0.66** | 0.50 | 0.88 | 0.004 |  |
|  | 31-35 | 914 | **79.1** | 63.9 | 89.0 | **0.56** | 0.41 | 0.75 | <0.001 |  |
|  | 36-49 | 899 | **77.1** | 64.0 | 86.4 | **0.49** | 0.36 | 0.66 | <0.001 |  |
| Mother's ethnicity* | |  |  |  |  |  |  |  |  | <0.001 |
| Mother's religion | Muslim | 3,164 | **79.8** | 57.7 | 91.9 | **0.89** | 0.71 | 1.12 | 0.330 | 0.015 |
|  | Catholic/Protestant | 1,971 | **84.7** | 75.1 | 91.1 | **1** | - | - | - |  |
|  | Animist/Atheist | 471 | **59.2** | 40.0 | 76.0 | **0.63** | 0.46 | 0.86 | 0.003 |  |
| Mother's education level | No education | 4,628 | **77.7** | 62.1 | 88.1 | **1** | - | - | - | <0.001 |
|  | Primary | 680 | **88.7** | 80.0 | 93.9 | **1.49** | 1.11 | 2.01 | 0.008 |  |
|  | Post primary | 298 | **91.9** | 86.8 | 95.2 | **2.17** | 1.34 | 3.51 | 0.002 |  |
| Mother's residence duration | Less than 3 years | 339 | **90.9** | 84.2 | 94.9 | **1** | - | - | - | 0.026 |
|  | 3 years or more | 5,267 | **79.1** | 64.1 | 88.9 | **0.62** | 0.41 | 0.96 | 0.032 |  |
| Mother's marital status | Single/Widow/Divorced | 120 | **85.0** | 73.5 | 92.0 | **0.94** | 0.53 | 1.67 | 0.838 | 0.191 |
|  | Monogamous union | 3,419 | **81.2** | 64.1 | 91.3 | **1** | - | - | - |  |
|  | Polygamous union | 2,038 | **77.0** | 63.6 | 86.5 | **0.85** | 0.72 | 1.01 | 0.068 |  |
| Parity | 1 | 1,020 | **87.5** | 76.6 | 93.8 | **1** | - | - | - | <0.001 |
| (stillbirths and live births) | 2 - 3 | 1,957 | **80.4** | 64.8 | 90.2 | **0.54** | 0.41 | 0.70 | <0.001 |  |
|  | 4 - 5 | 1,408 | **78.4** | 64.6 | 87.9 | **0.44** | 0.33 | 0.58 | <0.001 |  |
|  | 6 - 15 | 1,221 | **73.9** | 56.8 | 85.9 | **0.38** | 0.29 | 0.51 | <0.001 |  |
| * Data not shown to comply with the ethical requirement of the Burkina Faso MoH | | | | | | | |  |  |  |

Supplementary material, table 6: Cluster-adjusted associations with care seeking in a health facility for childhood illness

|  |  | Total | Care seeking (%) | 95% CI | | Cluster-adjusted analysis | | | | |
| --- | --- | --- | --- | --- | --- | --- | --- | --- | --- | --- |
|  |  |  |  |  |  | Odds Ratio | 95% CI | | p | Likelihood ratio test p value |
|  |  |  |  |  |  |  |  |  |  |  |
|  |  |  |  |  |  |  |  |  |  |  |
| Household wealth quintile | Poorest | 320 | **43.1** | 31.3 | 55.8 | **1** | - | - | - | 0.363 |
|  | 2nd quintile | 308 | **50.0** | 37.2 | 62.8 | **1.20** | 0.85 | 1.70 | 0.296 |  |
|  | 3rd quintile | 385 | **49.6** | 37.5 | 61.8 | **1.15** | 0.83 | 1.60 | 0.387 |  |
|  | 4th quintile | 374 | **52.9** | 43.3 | 62.3 | **1.32** | 0.94 | 1.83 | 0.105 |  |
|  | Least poor | 433 | **56.6** | 49.4 | 63.5 | **1.37** | 0.99 | 1.90 | 0.056 |  |
| Mother's age at interview (years) | 14-20 | 297 | **53.2** | 44.8 | 61.4 | **1** | - | - | - | 0.178 |
|  | 21-25 | 479 | **52.4** | 40.5 | 64.1 | **0.85** | 0.62 | 1.16 | 0.309 |  |
|  | 26-30 | 471 | **50.5** | 40.1 | 60.9 | **0.82** | 0.60 | 1.12 | 0.208 |  |
|  | 31-35 | 274 | **50.4** | 38.2 | 62.5 | **0.82** | 0.58 | 1.17 | 0.276 |  |
|  | 36-49 | 302 | **45.7** | 34.4 | 57.4 | **0.65** | 0.46 | 0.91 | 0.014 |  |
| Mother's ethnicity* | |  |  |  |  |  |  |  |  | 0.690 |
| Mother's religion | Muslim | 1,021 | **49.0** | 37.1 | 61.0 | **1.04** | 0.80 | 1.35 | 0.786 | 0.109 |
|  | Catholic/Protestant | 623 | **57.0** | 46.3 | 67.0 | **1** | - | - | - |  |
|  | Animist/Atheist | 179 | **38.0** | 28.4 | 48.6 | **0.68** | 0.46 | 1.02 | 0.061 |  |
| Mother's education level | No education | 1,499 | **49.0** | 39.0 | 59.1 | **1** | - | - | - | 0.016 |
|  | Primary | 226 | **55.8** | 44.6 | 66.4 | **1.34** | 0.99 | 1.81 | 0.060 |  |
|  | Post primary | 98 | **64.3** | 51.9 | 75.0 | **1.71** | 1.09 | 2.68 | 0.020 |  |
| Mother's residence duration | Less than 3 years | 122 | **57.4** | 39.7 | 73.4 | **1** | - | - | - | 0.382 |
|  | 3 years or more | 1,701 | **50.1** | 40.3 | 60.0 | **0.84** | 0.56 | 1.25 | 0.383 |  |
| Mother's marital status | Single/Widow/Divorced | 50 | **44.0** | 28.2 | 61.2 | **0.82** | 0.45 | 1.49 | 0.508 | 0.701 |
|  | Monogamous union | 1,089 | **50.4** | 39.3 | 61.5 | **1** | - | - | - |  |
|  | Polygamous union | 678 | **51.6** | 41.5 | 61.6 | **0.94** | 0.76 | 1.16 | 0.545 |  |
| Parity | 1 | 364 | **54.9** | 46.0 | 63.6 | **1** | - | - | - | 0.258 |
| (stillbirths and live births) | 2 - 3 | 590 | **51.2** | 40.0 | 62.3 | **0.92** | 0.70 | 1.22 | 0.576 |  |
|  | 4 - 5 | 446 | **50.4** | 39.0 | 61.8 | **0.81** | 0.61 | 1.10 | 0.176 |  |
|  | 6 - 15 | 423 | **46.3** | 35.7 | 57.3 | **0.76** | 0.56 | 1.03 | 0.076 |  |
| Child's age (months) | 0-5 | 233 | **43.8** | 33.5 | 54.6 | **1** | - | - | - | <0.001 |
|  | 6-11 | 410 | **59.3** | 48.6 | 69.1 | **1.82** | 1.29 | 2.58 | 0.001 |  |
|  | 12-23 | 641 | **54.4** | 44.4 | 64.2 | **1.55** | 1.12 | 2.15 | 0.008 |  |
|  | 24-59 | 553 | **43.2** | 32.3 | 54.8 | **1.00** | 0.72 | 1.40 | 0.994 |  |
| Child's sex | Boy | 954 | **51.9** | 42.8 | 60.8 | **1** | - | - | - | 0.072 |
|  | Girl | 867 | **49.3** | 38.6 | 59.9 | **0.83** | 0.68 | 1.02 | 0.072 |  |
| * Data not shown to comply with the ethical requirement of the Burkina Faso MoH | | | | | | | |  |  |  |

Supplementary material, table 7a: Distribution of distance to the closest facility in under-five children at risk during the study period

| Distance to the closest health facility (%) |  | < 2 km | 2 - 4 km | 4 - 7 km | > 7 km | Total | p value† |
| --- | --- | --- | --- | --- | --- | --- | --- |
| Household wealth quintile | Poorest | 22.8 | 21.1 | 25.4 | 30.7 | 29,738 | 0.697 |
|  | 2nd quintile | 24.0 | 22.3 | 24.2 | 29.5 | 33,590 |  |
|  | 3rd quintile | 25.0 | 21.6 | 23.1 | 30.3 | 37,588 |  |
|  | 4th quintile | 25.7 | 21.8 | 23.5 | 28.9 | 41,731 |  |
|  | Least poor | 27.6 | 20.2 | 23.7 | 28.6 | 49,304 |  |
| Mother's age at birth (years) | 14 - 20 | 24.4 | 19.9 | 23.8 | 31.9 | 43,588 | 0.015 |
|  | 21 - 25 | 25.1 | 20.8 | 23.8 | 30.3 | 53,765 |  |
|  | 26 - 30 | 25.1 | 21.5 | 24.2 | 29.2 | 43,982 |  |
|  | 31 - 35 | 25.6 | 22.7 | 24.0 | 27.7 | 30,967 |  |
|  | 36 - 49 | 27.2 | 22.5 | 23.7 | 26.7 | 21,906 |  |
| Mother's ethnicity* | |  |  |  |  |  | 0.005 |
| Mother's religion | Muslim | 27.9 | 22.3 | 22.4 | 27.4 | 109,106 | 0.110 |
|  | Catholic/Protestant | 25.4 | 22.2 | 26.2 | 26.2 | 65,153 |  |
|  | Animist/Atheist | 10.3 | 12.2 | 24.7 | 52.8 | 20,026 |  |
| Mother's education level | No education | 23.9 | 21.1 | 24.2 | 30.8 | 175,107 | < 0.001 |
|  | Primary | 35.7 | 22.1 | 22.6 | 19.6 | 15,203 |  |
|  | Post primary | 44.7 | 22.3 | 18.5 | 14.6 | 3,979 |  |
| Mother's marital status | Single/Widow/Divorced | 36.7 | 21.3 | 23.4 | 18.6 | 4,032 | 0.017 |
|  | Monogamous union | 27.6 | 20.9 | 24.5 | 27.0 | 104,433 |  |
|  | Polygamous union | 21.9 | 21.7 | 23.2 | 33.2 | 85,775 |  |
| Mother's residence duration | Less than 3 years | 32.9 | 25.6 | 20.9 | 20.6 | 6,026 | 0.059 |
|  | 3 years or more | 25.0 | 21.1 | 24.0 | 29.9 | 188,267 |  |
| Child's birth order (live births) | 1 | 26.8 | 21.2 | 23.3 | 28.8 | 36,594 | 0.008 |
|  | 2 - 3 | 26.1 | 21.4 | 23.5 | 29.1 | 65,228 |  |
|  | 4 - 5 | 25.2 | 21.5 | 24.2 | 29.2 | 48,321 |  |
|  | 6 - 17 | 23.0 | 20.8 | 24.8 | 31.5 | 44,150 |  |
| Child's birth interval length from previous live birth (excluding first birth) (months) | < 24 | 19.5 | 18.1 | 25.1 | 37.4 | 26,489 | < 0.001 |
|  | [24 - 36[ | 22.1 | 20.0 | 24.8 | 33.1 | 66,234 |  |
|  | [36 - 48[ | 27.2 | 23.1 | 23.8 | 25.9 | 41,123 |  |
|  | ≥ 48 | 34.9 | 25.2 | 21.4 | 18.6 | 23,853 |  |
| Child's birth interval length to next live birth (excluding last birth) (months) | < 24 | 18.8 | 17.7 | 25.3 | 38.1 | 20,533 | < 0.001 |
|  | 24 - 35 | 21.5 | 19.6 | 24.9 | 33.9 | 52,432 |  |
|  | 36 - 47 | 26.6 | 22.8 | 23.8 | 26.8 | 30,200 |  |
|  | ≥ 48 | 34.5 | 24.8 | 21.6 | 19.1 | 13,796 |  |
| Child's sex | Boy | 25.3 | 21.3 | 23.8 | 29.7 | 99,060 | 0.241 |
|  | Girl | 25.3 | 21.2 | 24.1 | 29.5 | 95,225 |  |
| Child's age (months) | <1 | 24.6 | 20.9 | 23.9 | 30.6 | 111,688 | 0.005 |
|  | 1-5 | 24.7 | 21.0 | 23.9 | 30.4 | 117,555 |  |
|  | 6 - 11 | 24.8 | 21.1 | 23.9 | 30.2 | 113,666 |  |
|  | 12 - 17 | 25.0 | 21.1 | 24.0 | 30.0 | 110,451 |  |
|  | 18 - 23 | 25.2 | 21.2 | 24.0 | 29.7 | 106,455 |  |
|  | 24 - 35 | 25.4 | 21.4 | 23.9 | 29.4 | 112,924 |  |
|  | 36 - 59 | 25.9 | 21.6 | 23.9 | 28.6 | 119,680 |  |
| † Chi-squared test accounting for clustering at cluster and village levels | | | | | |  |  |
| * Data not shown to comply with the ethical requirement of the Burkina Faso MoH | | | | | | |  |

Supplementary material, table 7b: Distribution of distance to the closest hospital in under-five children at risk during the study period

| Distance to the closest hospital (%) | | < 10 km | 10 - 20 km | > 20 km | Total | p value† |
| --- | --- | --- | --- | --- | --- | --- |
| Household wealth quintile | Poorest | 27.5 | 58.5 | 14.0 | 25,823 | 0.062 |
|  | 2nd quintile | 28.3 | 58.3 | 13.4 | 30,099 |  |
|  | 3rd quintile | 24.9 | 61.2 | 14.0 | 34,151 |  |
|  | 4th quintile | 24.1 | 60.8 | 15.2 | 38,444 |  |
|  | Least poor | 18.9 | 62.7 | 18.4 | 46,409 |  |
| Mother's age at birth (years) | 14 - 20 | 23.6 | 60.9 | 15.5 | 39,187 | 0.278 |
|  | 21 - 25 | 23.8 | 60.2 | 16.0 | 48,741 |  |
|  | 26 - 30 | 24.1 | 60.5 | 15.3 | 40,191 |  |
|  | 31 - 35 | 24.6 | 60.8 | 14.6 | 28,548 |  |
|  | 36 - 49 | 25.0 | 60.7 | 14.4 | 20,515 |  |
| Mother's ethnicity* | |  |  |  |  | 0.042 |
| Mother's religion | Muslim | 23.9 | 61.7 | 14.4 | 105,485 | 0.790 |
|  | Catholic/Protestant | 24.8 | 60.0 | 15.2 | 59,943 |  |
|  | Animist/Atheist | 22.3 | 53.4 | 24.3 | 11,806 |  |
| Mother's education level | No education | 22.9 | 61.4 | 15.7 | 158,675 | 0.001 |
|  | Primary | 34.3 | 53.4 | 12.3 | 14,694 |  |
|  | Post primary | 33.1 | 55.3 | 11.5 | 3,869 |  |
| Mother's marital status | Single/Widow/Divorced | 30.0 | 56.7 | 13.3 | 3,800 | 0.476 |
|  | Monogamous union | 24.9 | 59.3 | 15.8 | 97,730 |  |
|  | Polygamous union | 22.7 | 62.4 | 14.9 | 75,661 |  |
| Mother's residence duration | Less than 3 years | 29.6 | 61.9 | 8.5 | 5,903 | 0.072 |
|  | 3 years or more | 23.9 | 60.5 | 15.6 | 171,339 |  |
| Child's birth order (live births) | 1 | 23.9 | 60.6 | 15.5 | 33,526 | 0.758 |
|  | 2 - 3 | 24.3 | 60.5 | 15.2 | 59,816 |  |
|  | 4 - 5 | 24.3 | 60.7 | 14.9 | 44,135 |  |
|  | 6 - 17 | 23.8 | 60.4 | 15.8 | 39,765 |  |
| Child's birth interval length from previous live birth (excluding first birth) (months) | < 24 | 20.7 | 60.8 | 18.5 | 23,324 | 0.005 |
|  | 24 - 35 | 22.7 | 60.8 | 16.5 | 58,650 |  |
|  | 36 - 47 | 25.8 | 60.4 | 13.8 | 38,526 |  |
|  | ≥ 48 | 28.5 | 59.8 | 11.7 | 23,216 |  |
| Child's birth interval length to next live birth (excluding last birth) (months) | < 24 | 20.1 | 60.8 | 19.0 | 18,052 | 0.005 |
|  | 24 - 35 | 22.2 | 61.0 | 16.8 | 46,220 |  |
|  | 36 - 47 | 25.4 | 60.4 | 14.3 | 28,161 |  |
|  | ≥ 48 | 28.7 | 59.3 | 12.0 | 13,381 |  |
| Child's sex | Boy | 24.1 | 60.5 | 15.4 | 90,259 | 0.671 |
|  | Girl | 24.1 | 60.6 | 15.3 | 86,975 |  |
| Child's age (months) | <1 | 23.6 | 60.7 | 15.6 | 101,207 | 0.023 |
|  | 1-5 | 23.7 | 60.7 | 15.6 | 106,533 |  |
|  | 6 - 11 | 23.9 | 60.6 | 15.5 | 103,201 |  |
|  | 12 - 17 | 23.9 | 60.7 | 15.4 | 100,571 |  |
|  | 18 - 23 | 24.1 | 60.6 | 15.2 | 97,126 |  |
|  | 24 - 35 | 24.3 | 60.6 | 15.1 | 103,127 |  |
|  | 36 - 59 | 24.6 | 60.4 | 15.0 | 109,688 |  |
| † Chi-squared test accounting for clustering at cluster and village levels | | | | | |  |
| * Data not shown to comply with the ethical requirement of the Burkina Faso MoH | | | | | | |

Supplementary material, table 7c: Distribution of distance to the closest facility in mothers interviewed about their family behaviours

| Distance to the closest health facility (%) | | < 2 km | 2 - 4 km | 4 - 7 km | > 7 km | Total | p value† |
| --- | --- | --- | --- | --- | --- | --- | --- |
| Household wealth quintile | Poorest | 24.5 | 19.9 | 22.7 | 33.0 | 871 | 0.583 |
|  | 2nd quintile | 23.8 | 23.0 | 22.3 | 30.9 | 1,008 |  |
|  | 3rd quintile | 26.6 | 20.1 | 22.7 | 30.6 | 1,109 |  |
|  | 4th quintile | 29.5 | 20.7 | 23.4 | 26.4 | 1,222 |  |
|  | Least poor | 29.9 | 19.8 | 23.3 | 27.0 | 1,400 |  |
| Mother's age at interview (years) | 14-20 | 24.7 | 22.0 | 22.4 | 31.0 | 811 | 0.580 |
|  | 21-25 | 28.5 | 20.2 | 22.3 | 29.1 | 1603 |  |
|  | 26-30 | 27.3 | 19.6 | 22.9 | 30.2 | 1376 |  |
|  | 31-35 | 27.4 | 22.4 | 22.5 | 27.7 | 914 |  |
|  | 36-49 | 27.1 | 20.7 | 25.5 | 26.7 | 899 |  |
| Mother's ethnicity* | |  |  |  |  |  | 0.005 |
| Mother's religion | Muslim | 29.3 | 22.1 | 20.3 | 28.3 | 3,164 | 0.125 |
|  | Catholic/Protestant | 28.2 | 20.6 | 27.1 | 24.1 | 1,971 |  |
|  | Animist/Atheist | 9.3 | 11.7 | 24.0 | 55.0 | 471 |  |
| Mother's education level | No education | 24.8 | 20.5 | 23.5 | 31.2 | 4,628 | < 0.001 |
|  | Primary | 37.9 | 21.0 | 21.2 | 19.9 | 680 |  |
|  | Post primary | 40.3 | 23.2 | 19.1 | 17.5 | 298 |  |
| Mother's marital status | Single/Widow/Divorced | 33.3 | 23.3 | 19.2 | 24.2 | 120 | 0.016 |
|  | Monogamous union | 29.6 | 21.0 | 23.5 | 25.9 | 3,419 |  |
|  | Polygamous union | 22.7 | 19.9 | 22.5 | 34.9 | 2,038 |  |
| Mother's residence duration | Less than 3 years | 33.3 | 27.7 | 21.8 | 17.1 | 339 | 0.051 |
|  | 3 years or more | 26.8 | 20.3 | 23.1 | 29.8 | 5,267 |  |
| Parity (stillbirths and live births) | 1 | 30.0 | 21.6 | 21.4 | 27.1 | 1,020 | 0.050 |
|  | 2 - 3 | 29.2 | 21.0 | 21.4 | 28.4 | 1,957 |  |
|  | 4 - 5 | 27.3 | 20.3 | 23.9 | 28.4 | 1,408 |  |
|  | 6 - 15 | 21.5 | 20.1 | 25.9 | 32.5 | 1,221 |  |
| † Chi-squared test accounting for clustering at cluster and village levels | | | | | |  |  |
| * Data not shown to comply with the ethical requirement of the Burkina Faso MoH | | | | | | |  |

Supplementary material, table 8: Sensitivity analysis for the association of distance to the closest facility or hospital with child mortality

|  |  | Household wealth quintiles excluded | | | | | Education level excluded | | | | | Birth intervals excluded | | | | |
| --- | --- | --- | --- | --- | --- | --- | --- | --- | --- | --- | --- | --- | --- | --- | --- | --- |
|  |  | Rate Ratio | 95% CI | | p | Likelihood ratio test p value | Rate Ratio | 95% CI | | p | Likelihood ratio test p value | Rate Ratio | 95% CI | | p | Likelihood ratio test p value |
|  |  |  |  |  |  |  |  |  |  |  |  |  |  |  |  |  |
|  |  |  |  |  |  |  |  |  |  |  |  |  |  |  |  |  |
| **Early neonatal mortality** |  |  |  |  |  |  |  |  |  |  |  |  |  |  |  |  |
| Distance to the closest facility (km) | < 2 | **1** | - | - | - | 0.027 | **1** | - | - | - | 0.028 | **1** | - | - | - | 0.003 |
|  | 2 - 4 | **0.92** | 0.78 | 1.08 | 0.293 |  | **0.91** | 0.77 | 1.07 | 0.252 |  | **0.92** | 0.78 | 1.08 | 0.314 |  |
|  | 4 - 7 | **1.07** | 0.92 | 1.25 | 0.396 |  | **1.06** | 0.91 | 1.23 | 0.489 |  | **1.10** | 0.94 | 1.29 | 0.216 |  |
|  | > 7 | **1.18** | 1.00 | 1.40 | 0.046 |  | **1.18** | 1.00 | 1.39 | 0.055 |  | **1.26** | 1.06 | 1.49 | 0.007 |  |
| Distance to the closest hospital (excluding Kantchari cluster) (km) | < 10 | **1** | - | - | - | 0.220 | **1** | - | - | - | 0.303 | **1** | - | - | - | 0.222 |
|  | 10 - 20 | **1.13** | 0.98 | 1.29 | 0.085 |  | **1.11** | 0.97 | 1.28 | 0.130 |  | **1.13** | 0.98 | 1.30 | 0.086 |  |
|  | > 20 | **1.09** | 0.89 | 1.33 | 0.408 |  | **1.07** | 0.87 | 1.30 | 0.529 |  | **1.12** | 0.91 | 1.36 | 0.282 |  |
| **Late neonatal mortality** |  |  |  |  |  |  |  |  |  |  |  |  |  |  |  |  |
| Distance to the closest facility (km) | < 2 | **1** | - | - | - | 0.553 | **1** | - | - | - | 0.449 | **1** | - | - | - | 0.158 |
|  | 2 - 4 | **1.01** | 0.80 | 1.28 | 0.915 |  | **1.04** | 0.82 | 1.31 | 0.774 |  | **1.04** | 0.82 | 1.31 | 0.743 |  |
|  | 4 - 7 | **1.14** | 0.91 | 1.45 | 0.262 |  | **1.17** | 0.93 | 1.48 | 0.187 |  | **1.24** | 0.98 | 1.56 | 0.073 |  |
|  | > 7 | **1.15** | 0.90 | 1.49 | 0.266 |  | **1.19** | 0.92 | 1.54 | 0.183 |  | **1.27** | 0.99 | 1.64 | 0.062 |  |
| Distance to the closest hospital (excluding Kantchari cluster) (km) | < 10 | **1** | - | - | - | 0.137 | **1** | - | - | - | 0.156 | **1** | - | - | - | 0.092 |
|  | 10 - 20 | **0.88** | 0.72 | 1.08 | 0.235 |  | **0.89** | 0.73 | 1.09 | 0.277 |  | **0.91** | 0.74 | 1.11 | 0.349 |  |
|  | > 20 | **1.11** | 0.83 | 1.49 | 0.485 |  | **1.12** | 0.83 | 1.50 | 0.457 |  | **1.19** | 0.89 | 1.59 | 0.247 |  |
| **Neonatal mortality** |  |  |  |  |  |  |  |  |  |  |  |  |  |  |  |  |
| Distance to the closest facility (km) | < 2 | **1** | - | - | - | 0.014 | **1** | - | - | - | 0.013 | **1** | - | - | - | 0.001 |
|  | 2 - 4 | **0.94** | 0.82 | 1.08 | 0.402 |  | **0.94** | 0.82 | 1.08 | 0.412 |  | **0.95** | 0.83 | 1.09 | 0.483 |  |
|  | 4 - 7 | **1.09** | 0.95 | 1.25 | 0.198 |  | **1.09** | 0.95 | 1.25 | 0.205 |  | **1.15** | 1.00 | 1.31 | 0.050 |  |
|  | > 7 | **1.19** | 1.03 | 1.38 | 0.022 |  | **1.19** | 1.03 | 1.39 | 0.019 |  | **1.27** | 1.10 | 1.48 | 0.001 |  |
| Distance to the closest hospital (excluding Kantchari cluster) (km) | < 10 | **1** | - | - | - | 0.614 | **1** | - | - | - | 0.703 | **1** | - | - | - | 0.395 |
|  | 10 - 20 | **1.04** | 0.92 | 1.17 | 0.507 |  | **1.03** | 0.92 | 1.17 | 0.582 |  | **1.05** | 0.93 | 1.18 | 0.417 |  |
|  | > 20 | **1.09** | 0.92 | 1.30 | 0.329 |  | **1.08** | 0.90 | 1.28 | 0.404 |  | **1.13** | 0.95 | 1.35 | 0.172 |  |
| **Post-neonatal under-5 mortality** |  |  |  |  |  |  |  |  |  |  |  |  |  |  |  |  |
| Distance to the closest facility (km) | < 2 | **1** | - | - | - | 0.522 | **1** | - | - | - | 0.492 | **1** | - | - | - | 0.120 |
|  | 2 - 4 | **0.97** | 0.90 | 1.05 | 0.446 |  | **0.98** | 0.90 | 1.05 | 0.528 |  | **1.00** | 0.92 | 1.08 | 0.988 |  |
|  | 4 - 7 | **0.99** | 0.92 | 1.07 | 0.827 |  | **1.00** | 0.93 | 1.08 | 0.976 |  | **1.05** | 0.97 | 1.14 | 0.205 |  |
|  | > 7 | **1.03** | 0.95 | 1.12 | 0.487 |  | **1.04** | 0.95 | 1.14 | 0.365 |  | **1.10** | 1.01 | 1.20 | 0.038 |  |
| Distance to the closest hospital (excluding Kantchari cluster) (km) | < 10 | **1** | - | - | - | 0.311 | **1** | - | - | - | 0.150 | **1** | - | - | - | 0.033 |
|  | 10 - 20 | **1.05** | 0.98 | 1.13 | 0.132 |  | **1.07** | 1.00 | 1.14 | 0.053 |  | **1.10** | 1.02 | 1.17 | 0.010 |  |
|  | > 20 | **1.03** | 0.93 | 1.14 | 0.534 |  | **1.05** | 0.95 | 1.16 | 0.374 |  | **1.09** | 0.99 | 1.21 | 0.091 |  |
| **Under-5 child mortality** |  |  |  |  |  |  |  |  |  |  |  |  |  |  |  |  |
| Distance to the closest facility (km) | < 2 | **1** | - | - | - | 0.064 | **1** | - | - | - | 0.050 | **1** | - | - | - | 0.002 |
|  | 2 - 4 | **0.96** | 0.90 | 1.03 | 0.249 |  | **0.96** | 0.90 | 1.03 | 0.299 |  | **0.99** | 0.92 | 1.06 | 0.691 |  |
|  | 4 - 7 | **1.01** | 0.94 | 1.08 | 0.742 |  | **1.02** | 0.95 | 1.09 | 0.601 |  | **1.07** | 1.00 | 1.15 | 0.054 |  |
|  | > 7 | **1.06** | 0.98 | 1.15 | 0.119 |  | **1.07** | 0.99 | 1.16 | 0.079 |  | **1.13** | 1.05 | 1.23 | 0.002 |  |
| Distance to the closest hospital (excluding Kantchari cluster) (km) | < 10 | **1** | - | - | - | 0.304 | **1** | - | - | - | 0.171 | **1** | - | - | - | 0.027 |
|  | 10 - 20 | **1.05** | 0.99 | 1.11 | 0.123 |  | **1.06** | 1.00 | 1.13 | 0.061 |  | **1.09** | 1.02 | 1.16 | 0.009 |  |
|  | > 20 | **1.04** | 0.95 | 1.14 | 0.398 |  | **1.05** | 0.96 | 1.15 | 0.312 |  | **1.10** | 1.00 | 1.20 | 0.056 |  |

Supplementary material, table 9: Sensitivity analysis for the association of distance to the closest facility with care seeking behaviours

|  | Distance to the closest facility (km) | Household wealth quintiles excluded | | | | | Education level excluded | | | | |
| --- | --- | --- | --- | --- | --- | --- | --- | --- | --- | --- | --- |
|  |  | Odds Ratio | 95% CI | | p | Likelihood ratio test p value | Odds Ratio | 95% CI | | p | Likelihood ratio test p value |
|  |  |  |  |  |  |  |  |  |  |  |  |
|  |  |  |  |  |  |  |  |  |  |  |  |
| Modern contraception use | < 2 | **1** | - | - | - | < 0.001 | **1** | - | - | - | < 0.001 |
|  | 2 - 4 | **0.79** | 0.64 | 0.97 | 0.022 |  | **0.78** | 0.64 | 0.96 | 0.021 |  |
|  | 4 - 7 | **0.69** | 0.56 | 0.85 | 0.001 |  | **0.66** | 0.54 | 0.82 | <0.001 |  |
|  | > 7 | **0.59** | 0.46 | 0.75 | <0.001 |  | **0.58** | 0.45 | 0.74 | <0.001 |  |
| 4 or more ANC in a facility | < 2 | **1** | - | - | - | < 0.001 | **1** | - | - | - | < 0.001 |
|  | 2 - 4 | **0.84** | 0.69 | 1.01 | 0.069 |  | **0.84** | 0.69 | 1.02 | 0.073 |  |
|  | 4 - 7 | **0.67** | 0.55 | 0.82 | <0.001 |  | **0.67** | 0.55 | 0.82 | <0.001 |  |
|  | > 7 | **0.52** | 0.41 | 0.65 | <0.001 |  | **0.51** | 0.41 | 0.64 | <0.001 |  |
| Facility delivery | < 2 | **1** | - | - | - | < 0.001 | **1** | - | - | - | < 0.001 |
|  | 2 - 4 | **0.63** | 0.44 | 0.90 | 0.011 |  | **0.63** | 0.44 | 0.90 | 0.011 |  |
|  | 4 - 7 | **0.39** | 0.27 | 0.55 | <0.001 |  | **0.38** | 0.27 | 0.54 | <0.001 |  |
|  | > 7 | **0.23** | 0.16 | 0.33 | <0.001 |  | **0.22** | 0.15 | 0.32 | <0.001 |  |
| Care seeking for childhood illness in a facility | < 2 | **1** | - | - | - | 0.004 | **1** | - | - | - | 0.006 |
|  | 2 - 4 | **0.82** | 0.60 | 1.11 | 0.192 |  | **0.84** | 0.62 | 1.14 | 0.266 |  |
|  | 4 - 7 | **0.77** | 0.57 | 1.04 | 0.091 |  | **0.75** | 0.55 | 1.03 | 0.071 |  |
|  | > 7 | **0.52** | 0.36 | 0.73 | <0.001 |  | **0.52** | 0.36 | 0.75 | <0.001 |  |
